# Vietnamese Version of the Geriatric Depression Scale (30 Items): Translation, Cross-Cultural Adaptation, and Validation

**DOI:** 10.3390/geriatrics6040116

**Published:** 2021-12-12

**Authors:** Thong Van Nguyen, Kien Trung Nguyen, Phuong Minh Nguyen, Nghiem Minh Nguyen, Chi Lan Ly, Thang Nguyen, Minh Thi Tuyet Nguyen, Hoang Minh Le, Xuyen Thi Kim Nguyen, Nghi Huynh Phuong Duong, Richard C. Veith, Tuan Van Nguyen

**Affiliations:** 1 Department of Psychiatry, Hanoi Medical University, Hanoi 100000, Vietnam; nvthong@ctump.edu.vn; 2 Department of Psychiatry, Can Tho University of Medicine and Pharmacy, Can Tho City 900000, Vietnam; nguyenxuyen2697@gmail.com (X.T.K.N.); dgnghi1610@gmail.com (N.H.P.D.); 3 Department of Physiology, Can Tho University of Medicine and Pharmacy, Can Tho City 900000, Vietnam; ntkien@ctump.edu.vn; 4 Department of Pediatrics, Can Tho University of Medicine and Pharmacy, Can Tho City 900000, Vietnam; nmphuong@ctump.edu.vn; 5 Can Tho Central General Hospital, Can Tho City 900000, Vietnam; mnghiem999@gmail.com (N.M.N.); lylanchi83@gmail.com (C.L.L.); 6 Department of Pharmacology and Clinical Pharmacy, Can Tho University of Medicine and Pharmacy, Can Tho City 900000, Vietnam; nthang@ctump.edu.vn; 7 Foreign Language Department, Can Tho University of Medicine and Pharmacy, Can Tho City 900000, Vietnam; nttminh@ctump.edu.vn; 8 Department of Traditional Medicine, Can Tho University of Medicine and Pharmacy, Can Tho City 900000, Vietnam; lmhoang@ctump.edu.vn; 9 Department of Psychiatry and Behavioral Science, University of Washington, Seattle, WA 98195-6560, USA; rcv@uw.edu; 10 National Institute of Mental Health, Hanoi 100000, Vietnam

**Keywords:** adaptation, depression, geriatrics, questionnaire, translation, validation, Vietnamese

## Abstract

The proportion of geriatric depression recorded in Vietnam was 66.9%. Depression in older people is a risk factor for problems related to dementia, poor quality of life, and suicide. To have a good Vietnamese questionnaire for assessing geriatric depression, we conducted the study to translate and cross-culturally adapt the Geriatric Depression Scale—long-form with 30 items (GDS-30). The study has two steps. Step 1 is a translation of the GDS-30 scale. We followed the guideline by Beaton et al., (2000 & 2007). Firstly, two translators (informed and uninformed) translated the questionnaires. Secondly, the translations were synthesized. Thirdly, back translation was performed by two translators fluent in both Vietnamese and English but completely unknown of the original version of the scale and did not have medical expertise. Finally, seven experts reached a consensus on the pre-final Vietnamese version (GDS-30). Step 2 is a field test of the questionnaires on people 60 years or older. Then, we determined the internal consistency and test-retest reliability of the questionnaire in 55 Vietnamese inpatients in a geriatric department. Construct validity was determined by examining the relationship between depressive scores and patient characteristics. The Vietnamese version of GDS-30 was built with the agreement of all experts on the semantic, idiomatic, experiential, and conceptual equivalences between the original and pre-final Vietnamese versions of the GDS-30. The Cronbach’s alpha coefficient value was 0.928, indicating the items’ adequate internal consistency. Spearman’s correlation coefficient value of total scores between the first and second interviews showed medium correlation (0.479, *p* < 0.001), and the stability is acceptable. The GDS-30 scale reached the construct validity because the proportion of geriatric depression according to GDS-30 was significantly different between characteristics groups, such as gender, employment, level of education, economic status, and sleep disturbance. The Vietnamese version of the GDS-30 scale had high consistency, satisfactory reliability, and understanding and can be used as a screening tool for depression in elderly patients in primary healthcare centers. This is the first depression rating scale for the elderly in Vietnam to be translated and validated. Non-psychiatric health professionals or patients can quickly self-assess and screen for the illness.

## 1. Introduction

In 2020, the proportion of people aged 65 and over accounted for 9.3% of the total population, equivalent to 727 million people in the world [1]. In Vietnam, the proportion of older people (aged 60 years and older) has increased quite rapidly since the beginning of the 20th century, rising from 8.1% (in 1999) to 8.6% (in 2009) and reaching 11.9% in 2019. This rate has been projected to increase to 28.3% by 2050 [2,3,4]. Aging is associated with a number of factors that result in the deterioration of physical and mental health.

Depression is one of the most common mental disorders in the elderly [5]. In 2018, the proportion of geriatric depression recorded in Vietnam was 66.9% [6]. Older people with depression are at risk for several additional problems, such as dementia, poor quality of life, and suicide [7,8,9]. However, a 2012–2013 survey of 33,653 physician–patient encounters found less than 5% of adults were screened for depression in primary care [10]. Depression is popular in older adults, especially those with multimorbidity, such as thyroid disease, diabetes, heart disease, and other chronic medical conditions [11,12,13]. Depression and hypertension have also been shown to have interaction effects in a physiological way. In addition, depression could seriously affect their attitude of medication adherence, thereby decreasing their blood pressure control and quality of life, further aggravating the situation and creating a pathological spiral [14].

In 1982, Yesavage et al. developed the self-rated Geriatric Depression Scale (GDS) to screen for depression in the elderly [15]. A team of clinicians and researchers involved in geriatric psychiatry selected 100 questions believed to have the potential for distinguishing elderly depressives from normal subjects with various elements addressing cognitive complaints, motivation, future/past orientation, personal mood, etc. 30 questions with the highest correlation with the total score were chosen to create the GDS-30 scale [15]. The original version of this scale was written in English, and it has subsequently been translated and widely used in many Asian countries, such as India [16], Korea [17], and the Philippines [18].

The India version of this scale was conducted in the rural community of Ballabgarh in northern India with 1554 samples, mostly illiterate Hindi-speaking residents of Ballabgarh aged 55+. Although the large sample was selected, it was just distributed in a rural area, so the difference of depression in non-illiterate and illiterate people was unreliable [16]. The Korean version provided valid and reliable case-finding tools for screening major depression among the elderly psychiatric patients in Korea; however, the sample size of this study was not big enough to represent the Korean population [17]. The Philippines version of the GDS was performed in 505 elderly respondents who gave informed consent to participate in the study. Participants were required to be age 60 or older to comprehend both written and verbal English and Filipino and to exhibit no evidence of cognitive impairment. Respondents were required to complete all items from the English and the Filipino versions of the GDS. However, the result of this study was the GDS-15 (the short form of the original version), so it cannot fully evaluate different aspects of depression in the elderly [18].

Many popular depression rating scales have been translated into Vietnamese, such as the Center for Epidemiological Studies-Depression Scale (CES-D) [19], Patient Health Questionnaire—9 (PHQ-9) [20], and Zung Self-Rating Anxiety Scale (Zung SAS) [21]. However, these scales were not designed specifically for the elderly, so this study aims to translate and validate a Vietnamese version of the GDS-30 scale to screen for depression in the elderly.

## 2. Materials and Methods

### 2.1. Translation of the GDS-30 Scale

The GDS-30 scale was translated from the original English version of Yesavage et al. (1982) into a Vietnamese version [15]. It had 30 questions, including content related to depression of the elderly; the interviewees answered with two options (yes or no); depending on each question, the answer “yes” or “no” was counted as 1 point. Geriatric depression was assessed based on cumulative scores in two ways: (1) depression rating scores were divided into 2 groups, identified a depression (≥10 points) and no depression (<10 points); (2) depression rating scores were divided into 3 groups, identified as no depression (0–9 points), mild depression (10–19 points), and severe depression (20–30 points).

**Figure 1:**
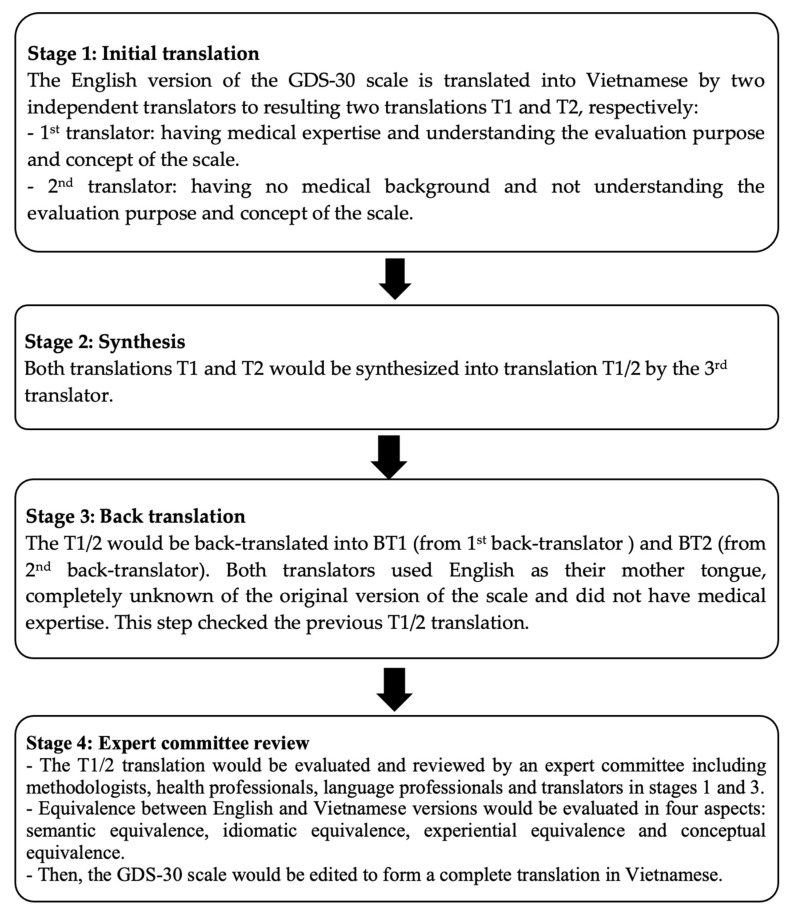
Translation and cross-cultural adaptation.

The GDS-30 scale was translated according to Beaton et al. (Figure 1) [22,23].

### 2.2. Validation of the GDS-30 Scale

#### 2.2.1. Study Design

The Vietnamese version of the GDS-30 scale was validated among elderly hypertensive inpatients (aged 60 years and above) at the Department of Geriatrics, Can Tho Central General Hospital, from May 2020 until March 2021. Sampling criteria included patients diagnosed with hypertension with systolic blood pressure ≥ 140 mmHg and/or diastolic blood pressure ≥ 90 mmHg [24] or were being treated for hypertension and agreed to participate in this study. Regarding the subject exclusion criteria in our study: any participant who has an acute illness, hearing impairment, language barrier, poor communication, a serious life problem within two weeks, and severe dementia were not included in our study.

#### 2.2.2. Data Collection

Participants were invited to a face-to-face interview through prepared questionnaires. We also conducted a second face-to-face interview on previously interviewed patients to assess the test-retest reliability, excluding patients discharged from the hospital. The length between the two interviews ranged from 2 weeks to a month [25]. This study conducted second interviews after 7–14 days (2 weeks) because of the limited time.

#### 2.2.3. Sample Size

The response rate of 1:10 (i.e., each question required 10 patients) was chosen to calculate the required sample size using established methods [26,27]. The GDS-30 scale had 30 questions, so the estimated sample size was 300 patients. An additional 10% were selected to accommodate lost participants during research time; the required sample size was 330 patients.

#### 2.2.4. Validation Criteria

##### Reliability

Internal consistency was estimated using Cronbach’s alpha coefficient. The scale was considered adequate internal consistency when the Cronbach’s alpha value was > 0.5 [28].

Test-retest was based on the repetition between results of the first and second interviews and evaluated using Spearman’s correlation coefficient. The Spearman’s coefficient value > 0.3 and *p*-value < 0.05 was considered good test-retest reliability [29,30].

#### 2.2.5. Validity

Content validity was based on the expert committee’s review and the equivalence score between the source and target version of the scale. Content validity was accepted when all questions received the consensus of more than half of the expert committee members.

Construct validity was evaluated by assessing the relationship between results of the first response and depression-related characteristics, such as age, gender, current occupation, education level, marital status, economic status, sleep disorder according to the Pittsburgh Sleep Quality Index (PSQI). The outcomes were considered statistically significant if the *p*-values were < 0.05.

#### 2.2.6. Data Analysis

Data were entered using Epidata 3.0 and were processed using SPSS version 18.0 (IBM Corp., New York, NY, USA). Descriptive statistics describe the proportions, frequencies of categorical variables, and the mean, standard deviation (SD) of continuous variables. The scale’s reliability was assessed by internal consistency (Cronbach’s alpha coefficient) and test-pretest (Spearman’s correlation coefficient). The chi-squared test with odds ratios (OR) and 95% confidence intervals (CIs) was applied to assess the relationship between depression and depression-related characteristics. The principal component analysis method (PCA) with varimax rotation was used to determine the factor structure of the GDS-30 scale.

#### 2.2.7. Ethics Approval

The Institutional Review Board approved this study for Ethics in Biomedical Research—Hanoi Medical University on 10 April 2020 (approval number: 72/GCN—HĐĐĐNCYSH—ĐHYHN). The purpose and content of the study were explained clearly and specifically to the participants, who signed a consent form. Participants also had the right to refuse to participate in the study without affecting treatment.

## 3. Results

The table showed the geriatric depression scale in the original English version and Vietnamese version after translation according to the above process (Table 1).

A total of 330 patients were selected (mean age = 75.2 ± 8.5, female = 229, 69.4%). Most patients were not employed (76.4%) and had primary school or lower (75.2%). There were 63.6% of the patients currently living with their spouses. Most of the patients were not poor (91.8%). Based on the PSQI scale, with a cut-off point of 5, patients’ percentage of sleep disturbance was 82.7%. Mean GDS point: 9.34, SD: 7.65. The proportion of participants with depression according to the GDS scale (score ≥10) was 41.5% (Table 2).

### 3.1. Reliability Test

#### 3.1.1. Consistency

The Cronbach’s alpha coefficient value was 0.928, indicating the items’ adequate internal consistency (Table 3).

#### 3.1.2. Stability

Spearman’s correlation coefficient value of total score between the first and second interviews after 2 weeks showed a satisfactory correlation (0.479, *p* < 0.001). In the GDS-30 scale, 6 items had no correlation (*p* > 0.05), 11 items had weak correlation (<0.4) and 13 items had medium correlation (0.4–0.6). (Table 4).

### 3.2. Validity

#### 3.2.1. Content Validity

Between the source and target version of the GDS-30 scale, the expert committee assessed the average score of 0.84 points for experience equivalence. The other criteria (semantic, idiomatic, and conceptual equivalence) achieved an average score of 0.88 (Table 5).

#### 3.2.2. Construct Validity

There was a significant difference in the rates of depression between gender, employment, education level, economic status, depression diagnosed according to ICD-10 and sleep disturbance according to PSQI (Table 6).

The factor loadings ranged from 0.40 to 0.8. The factorial analysis produced 5 factors for the GDS that represents 52.874% of the variance (Table 7).

The first factor was composed of 7 items: 7, 8, 6, 13, 9, 4, 16. This contributed to 33.57% of the total variance. This factor was called “sad mood”.

The second factor was composed of 4 items: 15, 5, 19, 21. This contributed to 5.73% of the total variance. This factor was called “positive mood”.

The third factor was composed of 5 items: 23, 24, 18, 25, 17. This contributed to 4.62% of the total variance. This factor was called “agitation and pessimistic”

The fourth factor was composed of 3 items: 12, 28, 2. This contributed to 4.62% of the total variance. This factor was called “social withdrawal”.

The fifth factor was composed of 2 items: 14, 30. This contributed to 3.88% of the total variance. This factor was called “cognitive inefficiency”.

## 4. Discussion

This study aimed to translate and validate the first Vietnamese version of the GDS-30 scale for people over 60 years of age. The results showed that this scale could be a reliable screening tool for geriatric depression in Vietnam. Our findings are consistent with previous studies that have demonstrated that this scale can be used effectively to screen for depression in the elderly in many countries worldwide with different versions of the number of items [31,32].

Early diagnosis of depression in the elderly is essential. In our study, the proportion of elderly participants identified with depression was 41.5% according to the GDS-30 scale (≥10 points), a finding that is consistent with rates of depression in the elderly in many studies worldwide, which vary from 20.7 to 53.8% [33,34,35,36] among different countries and using varying diagnostic approaches

In our study, the Cronbach’s alpha coefficient value was 0.928, which showed very good internal consistency of the items [28]. All items had a suitable composite coefficient of correlation (≥0.3). “Cronbach’s alpha coefficient value if items were deleted” of all items was lower or equal to Cronbach’s alpha coefficient value of this scale, so no item was excluded from the scale. Spearman’s correlation coefficient between the two interviews showed a medium correlation (0.479 with *p* < 0.001) regarding scale stability. After the first and second interviews, there were 6 items with no stability, including 3 positive items (1, 15, 19) and 3 negative items (3, 23, 26). Therefore, the patient’s emotions were not affected by their response. This study also determined the efficacy of the Vietnamese version of the GDS-30 scale compared with the ICD-10 (gold-standard) in diagnosing depression in older adults. The GDS-30 scale reached construct validity because the proportion of geriatric depression according to GDS-30 was significantly different between characteristics groups.

Previous validation studies also illustrated the high consistency of the GDS-30 scale. In the Netherlands version, the Cronbach’s alpha coefficient value was 0.88 [37] and showed a good level of consistency [28]. For other language versions, Cronbach’s alpha coefficient values ranged from 0.839 to 0.91 [38,39]. These data showed that the Vietnamese version of the GDS-30 scale was reliable and had adequate internal consistency.

In the Korean version, the test-retest reliability (Pearson correlation) was 0.91 (*p* < 0.01), indicating that the performance of the GDS-30 is highly stable over time [40]. In the Italian version, the reliability of scale after re-testing (ICC) was 0.91 [39]. Period conducted a re-test in the two studies mentioned above ranged from 1–7 days, while our study conducted a re-test in 7–14 days. The older patients could not remember exactly what happened in the past. Therefore, Spearman’s correlation coefficient value in this study was average.

In this study, the Vietnamese version of the GDS-30 scale produced 5 factors (sad mood, positive mood, agitation and pessimism, social withdrawal, cognitive inefficiency). This was similar to the original English version of the GDS, including sad mood (8, 6, 23, 13, 16, 18, 10. 24, 22), lack of energy (29, 20. 21, 30, 25, 2), positive mood (15, 27, 9, 5, 7, 19), agitation (24, 11, 4) and social withdrawal (12, 28) [41]. In the original version, 4 items (1, 3, 14, 17) were absent. The Korean version included 5 factors: sad mood and agitation (6, 18, 11, 8, 13, 24, 16, 25, 10. 3), positive mood (1, 9, 7, 15, 19, 22, 27, 5, 23), lack of energy (2, 21, 20, 17), cognitive inefficiency (14, 26, 30), and social withdrawal (12, 28) [40]. In the Italian version, the scale was divided into 5 factors: sad mood and agitation (6, 8, 10. 11, 16, 22, 24, 25), cognitive inefficiency (1, 3, 15, 17, 19, 23), lack of energy (2, 20. 21, 29), positive mood (5, 7, 9, 27), social withdrawal (4, 12, 13, 14, 18, 26, 28) [39]. The different versions were similar in most factors, such as sad mood, agitation, social withdrawal, positive mood, cognitive inefficiency. In addition, because of a factor score of less than 0.5, nine items (1, 3, 10, 11, 20, 22, 26, 27, 29) did not belong to any factors in this study that was different from the English, Korean, or Italian versions. This was a new finding in our study.

Our study proved that the Vietnamese version of the GDS-30 scale had high consistency, satisfactory reliability, understanding. It can be used as a screening tool for depression in elderly patients in primary healthcare centers. Non-psychiatric health professionals or patients can quickly self-assess and screen for depressive symptoms. However, because of time limitation, our study could not be conducted in the community. Assessing the sensitivity and specificity of the Vietnamese version of the GDS-30 also needs to be considered.

## 5. Conclusions

This is the first depression rating scale for the elderly in Vietnam to be translated and validated. The Vietnamese version of the GDS-30 scale had high consistency, satisfactory reliability, and clarity. This study was conducted in a hospital and given the complexities of the population in this setting; future studies conducted among outpatients would be useful in further clarifying the factor analysis aspects of the Vietnamese translated GDS-30 items. Future studies will also be needed to assess the sensitivity and specificity of the Vietnamese version of the GDS-30. Still, it appears that the Vietnamese version of the GDS-30 can be used as a screening tool for depression in elderly patients in primary healthcare centers by non-psychiatric health professionals or patients to quickly self-assess and screen for depressive illness.

## Figures and Tables

**Table 1:** The original English version and the Vietnamese version of the GDS.

Item	The Original English Version	Vietnamese Version
1 *	Are you basically satisfied with your life?	Về cơ bản Ông/Bà hài lòng với cuộc sống của mình?
2	Have you dropped many of your activities and interests?	Ông/Bà đã từ bỏ nhiều hoạt động và thú vui?
3	Do you feel that your life is empty?	Ông/Bà cảm thấy cuộc sống của mình thật trống rỗng?
4	Do you often get bored?	Ông/Bà thường cảm thấy buồn chán?
5 *	Are you hopeful about your future?	Ông/Bà thấy hy vọng vào tương lai của mình?
6	Are you bothered by thoughts you can’t get out of your head?	Ông/Bà có phiền muộn bởi những suy nghĩ trong đầu không thể bỏ được?
7 *	Are you good in spirits most of the time?	Tinh thần của ông bà tốt trong hầu hết thời gian?
8	Are you afraid that something bad is going to happen to you?	Ông/Bà có sợ điều gì đó không hay sắp xảy đến với mình?
9 *	Do you feel happy most of the time?	Hầu hết thời gian Ông/Bà cảm thấy hạnh phúc?
10	Do you often feel helpless?	Ông/Bà thường cảm thấy bất lực?
11	Do you often get restless and fidgety?	Ông/Bà thường cảm thấy bồn chồn, đứng ngồi không yên?
12	Do you prefer to stay at home, rather than going out and doing new things?	Ông/Bà thích ở nhà hơn ra ngoài và làm những điều mới mẻ?
13	Do you frequently worry about your future?	Ông/Bà thường xuyên lo lắng về tương lai?
14	Do you feel you have more problems with memory than most?	Ông/Bà cảm thấy mình có nhiều vấn đề về trí nhớ hơn hết?
15 *	Do you think it is wonderful to be alive now?	Ông/Bà nghĩ hiện tại được sống là tuyệt vời?
16	Do you often feel downhearted and blue?	Ông/Bà thường cảm thấy buồn và nản chí?
17	Do you feel pretty worthless the way you are now?	Theo tình trạng hiện giờ, Ông/Bà cảm thấy khá vô ích?
18	Do you worry a lot about the past?	Ông/Bà lo lắng nhiều về quá khứ?
19 *	Do you find life very exciting?	Ông/Bà nhận thấy cuộc sống rất hào hứng?
20	Is it hard for you to get started on new projects?	Ông/Bà thấy khó khăn để bắt đầu những dự định mới?
21 *	Do you feel full of energy?	Ông/Bà cảm thấy tràn đầy năng lượng?
22	Do you feel that your situation is hopeless?	Ông/Bà cảm thấy tình trạng của mình là không có hy vọng?
23	Do you think that most people are better off than you are?	Ông/Bà nghĩ hầu hết mọi người đều sung sướng hơn mình?
24	Do you frequently get upset over little things?	Ông/Bà thường thấy bực bội với những việc nhỏ nhặt?
25	Do you frequently feel like crying?	Ông/Bà thường cảm thấy muốn khóc?
26	Do you have trouble concentrating?	Ông/Bà có khó tập trung?
27 *	Do you enjoy getting up in the morning?	Ông /Bà có hào hứng thức dậy vào buổi sáng?
28	Do you prefer to avoid social gatherings?	Ông/Bà muốn tránh các tụ họp đông người?
29 *	Is it easy for you to make decisions?	Có dễ dàng để Ông/Bà đưa ra các quyết định?
30 *	Is your mind as clear as it used to be?	Ông/Bà vẫn minh mẫn như trước kia?

Scoring method: 1 point for the answer “Yes”, only for items marked with (*), 1 point for the answer “No”. The total score for all items would be converted to the GDS30 depressive cut-off points. No depression: 0–9 points, Depression: >9 points.

**Table 2:** Study population characteristics (*n* = 330).

		Frequency (*n*)	Percentage (%)
Age	Mean ± SD	75.24 ± 8.46	
Age group	60–69 years old	93	28.2
	70–79 years old	126	38.2
	≥ 80 years old	111	33.6
Gender	Female	101	30.6
	Males	229	69.4
Employment	Trade	13	3.9
	Farmer	41	12.4
	Housewife	21	6.4
	Stop working	252	76.4
	Other	3	0.9
Level of education	≤ Primary	248	75.2
	Secondary school	50	15.2
	High school	18	5.5
	> High school	14	4.2
Marital status	Married	210	63.6
	Single	6	1.8
	Divorced/separated/widowed	114	34.5
Economic status	Poor	27	8.2
	No poor	303	91.8
Sleep disturbance	Yes	273	82.7
	No	57	17.3

**Table 3:** Cronbach’s alpha coefficient of the GDS-30 scale (*n* = 330).

Item	Corrected Item-Total Correlation	Cronbach’s Alpha If the Item Was Deleted	Cronbach’s Alpha Coefficient
1	0.601	0.925	0.928
2	0.502	0.926	
3	0.585	0.925	
4	0.722	0.923	
5	0.480	0.926	
6	0.512	0.926	
7	0.645	0.924	
8	0.503	0.926	
9	0.641	0.924	
10	0.641	0.924	
11	0.555	0.925	
12	0.319	0.929	
13	0.558	0.925	
14	0.336	0.928	
15	0.526	0.926	
16	0.713	0.923	
17	0.536	0.925	
18	0.350	0.928	
19	0.636	0.924	
20	0.579	0.925	
21	0.592	0.925	
22	0.415	0.927	
23	0.474	0.926	
24	0.466	0.926	
25	0.519	0.926	
26	0.501	0.926	
27	0.528	0.926	
28	0.556	0.925	
29	0.569	0.925	
30	0.317	0.928	

**Table 4:** Spearman’s correlation coefficient of the GDS-30 scale (*n* = 55).

Item	Spearman’s Correlation Coefficient	*p*
1	0.152	0.268
2	0.386	0.004
3	0.126	0.359
4	0.439	0.001
5	0.297	0.028
6	0.458	<0.001
7	0.429	<0.001
8	0.421	0.001
9	0.443	0.001
10	0.452	0.001
11	0.331	0.014
12	0.603	<0.001
13	0.608	<0.001
14	0.346	0.01
15	−0.098	0.477
16	0.363	0.007
17	0.270	0.046
18	0.444	0.001
19	0.102	0.458
20	0.336	0.012
21	0.495	<0.001
22	0.313	0.02
23	0.179	0.191
24	0.373	0.005
25	0.530	<0.001
26	0.149	0.279
27	0.39	0.003
28	0.288	0.033
29	0.535	<0.001
30	0.542	<0.001
Total	0.479	<0.001

**Table 5:** Expert committee assessment score for GDS-30 scale.

Item	Semantic Equivalence	Idiomatic Equivalence	Experience Equivalence	Conceptual Equivalence
Scale title	7/7	7/7	7/7	7/7
1	7/7	7/7	7/7	7/7
2	6/7	7/7	7/7	7/7
3	7/7	7/7	6/7	7/7
4	7/7	7/7	7/7	7/7
5	5/7	5/7	5/7	5/7
6	6/7	6/7	5/7	6/7
7	7/7	7/7	6/7	7/7
8	5/7	5/7	5/7	5/7
9	7/7	7/7	7/7	7/7
10	5/7	5/7	5/7	5/7
11	4/7	4/7	4/7	4/7
12	7/7	7/7	7/7	7/7
13	7/7	7/7	7/7	7/7
14	5/7	5/7	4/7	5/7
15	6/7	6/7	6/7	6/7
16	6/7	6/7	6/7	6/7
17	5/7	5/7	4/7	5/7
18	7/7	7/7	7/7	7/7
19	5/7	5/7	5/7	5/7
20	7/7	7/7	7/7	7/7
21	7/7	7/7	7/7	7/7
22	7/7	7/7	7/7	7/7
23	4/7	4/7	4/7	4/7
24	7/7	7/7	7/7	7/7
25	7/7	7/7	7/7	7/7
26	7/7	7/7	7/7	7/7
27	5/7	5/7	5/7	5/7
28	5/7	5/7	4/7	5/7
29	7/7	7/7	6/7	7/7
30	7/7	7/7	6/7	7/7
Average score	184/210 (0.88)	185/210 (0.88)	177/210 (0.84)	185/210 (0.88)

**Table 6:** The relationship between the general characteristics and depression.

Characteristics		Depression		Non-Depression		*p*
		*n*	%	*n*	%	
Age group	60–69 years	30	32.3	63	67.7	0.102
	70–79 years	57	45.2	69	54.8	
	≥ 80 years	50	45.0	61	55.0	
Gender	Female	112	48.9	117	51.1	<0.001
	Male	25	24.8	76	75.2	
Employment	No	115	45.6	137	54.4	0.006
	Yes	22	28.2	56	71.8	
Level of education	Lower high school	132	44.3	166	55.7	0.002
	High school or higher	5	15.6	27	84.4	
Marital status	Other	52	43.3	68	56.7	0.612
	Marital	85	40.5	125	59.5	
Economic status	Poor	19	70.4	8	29.6	0.001
	No poor	118	38.9	185	61.1	
Sleep disturbance	Yes	131	48.0	142	52.0	<0.001
	No	6	10.5	51	89.5	

**Table 7:** Factor analysis of the GDS-30 scale.

Item	Factor				
	1	2	3	4	5
7. Are you in good spirits most of the time?	0.748				
8. Are you afraid that something bad is going to happen to you?	0.671				
6. Are you bothered by thoughts you can’t get out of your head?	0.664				
13. Do you frequently worry about the future?	0.658				
9. Do you feel happy most of the time?	0.653				
4. Do you often get bored?	0.542				
16. Do you feel downhearted and blue?	0.531				
15. Do you think it is wonderful to be alive now?		0.687			
5. Are you hopeful about the future?		0.667			
19. Do you find life very exciting?		0.653			
21. Do you feel full of energy?		0.615			
23. Do you think that most people are better off than you are?			0.708		
24. Do you frequently get upset over little things?			0.664		
18. Do you worry a lot about the past?			0.556		
25. Do you frequently feel like crying?			0.526		
17. Do you feel pretty worthless the way you are now?			0.510		
12. Do you prefer to stay at home, rather than going out and doing new things?				0.746	
28. Do you prefer to avoid social gatherings?				0.698	
2. Have you dropped many of your activities and interests?				0.562	
30. Is your mind as clear as it used to be?					0.795
14. Do you feel you have more problems with memory than most?					0.792
Percent Variance (%)	33.57	5.73	5.07	4.62	3.88

## Data Availability

Not applicable.
